# Chemically reacted blood Cu O nanofluid flow through a non-Darcy porous media with radially varying viscosity

**DOI:** 10.1038/s41598-023-48692-3

**Published:** 2024-01-18

**Authors:** Mahmoud E. Ouaf, M. Y. Abouzeid

**Affiliations:** Department of Mathematics, Faculty of Education, Ain Shams University, Heliopolis, Cairo, Egypt

**Keywords:** Fluid dynamics, Applied mathematics

## Abstract

The study investigates the flow of a Newtonian Cu O nanofluid through a non-Darcy porous medium with radially varying viscosity, which is crucial for various industries such as pharmaceuticals, chemicals, nuclear, solar, and solar technologies. The peristaltic motion of the nanofluid is studied with thermal radiation and chemical reaction effects, and the viscosity varies with both radius and axial coordinates. The study assumes low Reynolds and long wavelength assumptions and uses the homotopy perturbation technique to obtain a semi-analytical solution of velocity, temperature, nanoparticle concentration, and skin friction. The results show that axial velocity increases with the increase of slip velocity and viscosity parameters, while wave amplitude and chemical reaction parameters increase while nanoparticle concentration decreases. High viscosity parameters allow fluid nanoparticles to gain more active energy and move more freely, which is the main idea behind crude oil refinement. This physical modeling is essential for physiological flows, such as stomach juice flow during endoscope insertion.

## Introduction

Peristaltic pumping is a method of moving fluid through a distensible tube, used to propel food through the esophagus and move small blood vessels in living systems. Engineers have developed pumps for industrial and medical applications using this concept. Latham^1^ initiated a subject with basic mathematical assumptions, including constant viscosity, Newtonian fluid, symmetrical membrane, and an infinitely long wavelength approximation. Over time, these assumptions were modified to become more realistic. Peristaltic pumping devices have been utilized in the nuclear sector to transport various materials, such as slurries, delicate or caustic fluids, hygienic fluids, and toxic fluids. Peristaltically controlled micro-electro-mechanical system devices enable fluid conveyance in specific situations without the need to move internal mechanical parts^2^. A study of peristalsis in fluid mechanics has gained prominence in the past three decades due to its relevance to biological systems and industrial applications. Blood, for example, is a suspension of red, white, and platelet cells in plasma. Bums and Parkes^3^ studied peristaltic motions using the Stokes equation and assumed large wavelengths. However, their results only apply to slow creeping fluid motion, which corresponds to very slow physiology. Our results also apply to motion with very low frequency. Mansour and Abou-Zeid^4^ investigated the subject of heat and mass transfer effects on non-Newtonian fluid flow in a non-uniform vertical tube with peristalsis. The obtained data indicate that the temperature increases as the Eckert number (Ec) and the Weisseing number (Wi) increase. Conversely, a behavior that is opposite to the temperature behavior is observed for concentration. Furthermore, many studies of the peristaltic flow of Newtonian or non-Newtonian fluids have been published^5–14^.

Over the past two decades, "nanofluids" have emerged as a result of the convergence of thermal engineering and nanotechnology research. These heat transfer fluids contain nanoparticles ranging from 1 to 100 nm, dispersed throughout the base fluid. The size of the nanoparticles must be less than 100 nm to maximize fluid stability. Common base fluids include water, oils, organic liquids, and biological fluids. Nanoparticles contain nonmetals like Al_2_O_3_, Fe_2_O_3_, and Cu O, as well as metals like Ag, Cu, and Al^15,16^. Over the years, numerous studies have enhanced our understanding of nanofluids, documenting various preparation techniques and their heat transfer capacities. Mostafa et al.^17^ investigate the the impact of Cattaneo-Christov heat flux of an incompressible flow which obeys Carreau nanofluid inside a symmetric channel in the existence of the porous medium. It is found that the elevate in the slip velocity parameter dwindle the velocity. Meanwhile, the rise in the value of thermal relaxation time parameter led to decay the temperature of the fluid. Abuiyada et al.^18^ has studied the problem of the peristaltic motion of Bingham plastic nanofluid through a vertical symmetric channel. It has been found that increases in the Weissenberg, Reynolds, Forschheimer, and Biot numbers result in an increase in the concentration of nanoparticles. The issue of the effect of viscous dissipation and radiation on peristaltic mixed convection slip flow of a Bingham nanofluid through a non-darcy porous medium in an inclined non-uniform duct with has been discussed by Eldabe et al.^19^. It is clear that when the velocity ratio B increases, the temperature decreases, whereas Eckert's number increases with temperature. Nanofluids can be studied theoretically or experimentally to control heat transfer processes. Two-phase simulations are used: single-phase, where nanoparticles are treated as a pure fluid, and two-phase, where slip velocities exist between nanoparticles and fluid molecules. The two-phase model considers variable concentrations of nanoparticles in mixtures, and the velocity between fluid and particles may not vanish due to factors like friction, Brownian forces, gravity, Brownian diffusion, thermophoresis properties, and dispersion. Several studies^20–23^ have been conducted, particularly on peristalsis in nanofluids. In the present study, we assume the two-phase model, i.e., both Brownian and thermophoresis effects will appear in the governing equation system^24^.

Darcy's law is a fundamental concept in understanding flow dynamics in reservoirs, indicating a linear relationship between volumetric flow rate and pressure gradient. It is used in various mechanical and industrial processes, such as underground water purification, oil recovery, and pipe development. Darcy's model was initially developed for weak porosity conditions and lower velocities. It was then modified by Forchheimer^25^, utilizing a nonlinear factor through velocity, and the new name for this model was the Darcy-Forchheimer model. Homogeneous fluid flow through a Darcy medium was described by Muskat^26^. Analytic treatment for electrical MHD non-Newtonian fluid flow over a stretching sheet through a porous medium is an issue that Adam^27^ has researched. It has been noted that the chemical reaction and porous matrix with moderate magnetic parameters reduce temperature and concentration fields in the flow domain, while the interaction of the magnetic field counters the velocity and concentration distribution. An analytic solution for electrical magneto-hydrodynamic Darcy-Forchheimer three-dimensional non-Newtonian nanofluid flow with convective boundary conditions was investigated by Adam^28^. It has been discovered that the radiation, Biot number, and thermophoresis Brownian motion features all induce an increase in the temperature profile. The concentration profile rises with higher Biot numbers and the thermophoresis parameter but falls with the Brownian motion parameter. Due to its usage in science and technology, including the generation of crude oil, fermentation processes, nuclear waste disposal, microelectronic devices, and hemodialyzers, nanofluid flow through porous media has garnered a lot of attention. Even though Darcy’s Law has only been applied to reservoir studies, there is a substantial body of evidence indicating high-velocity non-Darcy flow occurs in oil and gas reservoirs. Examples include flow in the formation near oil or gas production, groundwater pumping, and liquid waste injection wells. These articles^29–34^ look at some interesting non-Darcy flow results.

Eldabe et al.^35^ examined the action of varying viscosity on the peristaltic flow of a Newtonian fluid via a tube. They also investigated the tube’s centerline trapping phenomenon. We aim in the present study to extend the work of Eldabe et al.^35^ to include heat transfer, non-Darcian effects, and both thermophoresis and Brownian features. So, the fundamental target of this study is to investigate the impacts of the Forchheimer effect on the MHD peristaltic motion of a Newtonian nanofluid with radially and axially varying viscosity. The fluid is flowing through a co-axial horizontal tube. The effects of thermal radiation, heat sources, and chemical reactions are also included. Moreover, the slip condition impacts the axial velocity distributions. The use of a low Reynolds number and long wavelength assumptions can reduce the mathematical complexity of our investigation. By combining the homotopy perturbation approach and the traditional perturbation method, these non-linear equations are analytically disbanded. A set of graphs is used to graphically assess and show the numerical effects of various physical characteristics on the different distributions. Physically, our model refers to the food transport downward from the esophagus to the stomach and breezes the motion of chyme (food semblance after digestion) across the small intestine.

## Problem formulation

**Figure 1:**
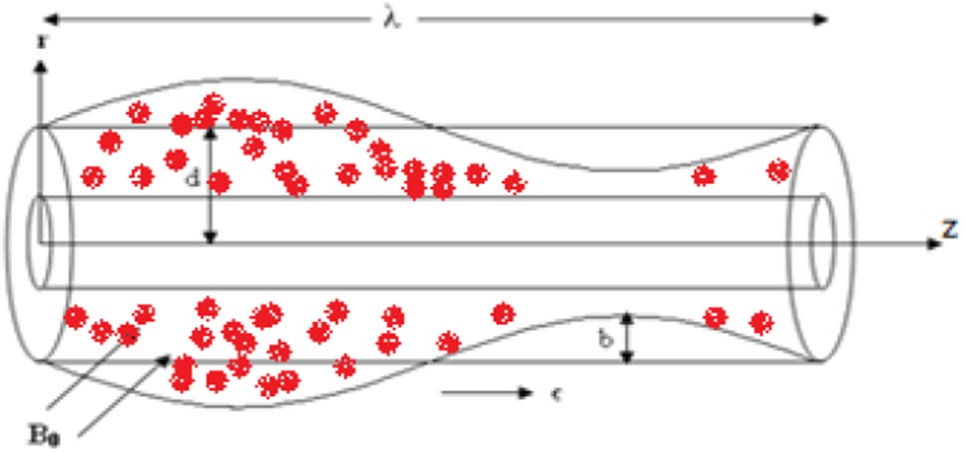
Schematic of the model^39^.

We investigated blood copper oxide Cu O nanofluid fluid flow through a porous medium between two co-axial tubes. A uniform transverse magnetic field (B_0_) is assumed to exist in the fluid. In contrast to the outer tube, which has a sinusoidal wave moving down its wall, the inner tube is rigid and uniform (see Fig. 1). Moreover, the nanoparticles Cu O are used with the base fluid to shape nanofluid. Table 1 analyzes the thermal properties of Cu O-blood nanofluid^36–38^.

**Table 1:** Thermophysical properties of base fluid (blood) and Cu O nanoparticles.

Physical properties	Fluid phase (blood)	Cu O	Fluid phase (water)
$$C_{p} $$ (J/kg K)	3617	540	4179
ρ (kg/m^3^)	1050	6500	997.1
k (W/m K)	0.52	18	0.613
$\beta \times 10^{ - 5} \;\left( {1/{\text{K}}} \right)$	37	0.85	21
$D_{p}$ (nm)	0.58	29	0.384

The cylindrical coordinate system (*r*, *θ*, *z*) is used. The inner and outer tube equations are:

1 $$ r_{{1}} = a,\;\;r_{2} = H = d + b\sin \frac{2\pi }{\lambda }(z - ct) $$

For an incompressible flow in the fixed wave and under consideration of the mass flux due to the energy gradient and the revised Buongiorno's nanofluid model depending on concentration, the governing equations are as follows^34^:

2 $$ \frac{\partial u}{{\partial r}} + \frac{u}{r} + \frac{\partial w}{{\partial z}} = 0, $$

3 $$ \begin{gathered} \rho \,\left( {u\frac{\partial u}{{\partial r}} + w\frac{\partial u}{{\partial z}}} \right) = - \frac{\partial P}{{\partial r}} + \frac{\partial }{\partial r}\left( {2\mu (r,\,z)\frac{\partial u}{{\partial r}}} \right) + \frac{\partial }{\partial z}\left( {\mu (r,\,z)\left( {\frac{\partial u}{{\partial z}} + \frac{\partial w}{{\partial r}}} \right)} \right) \hfill \\ \quad \quad \quad \quad \quad \quad \quad \quad \quad - \,\frac{2\mu (r,\,z)}{r}\left( {\frac{\partial u}{{\partial r}} - \frac{u}{r}} \right) - \left( {\sigma B_{0}^{2} + \frac{\mu (r,\,z)}{K}} \right)u + C^{ * } u^{2} , \hfill \\ \end{gathered} $$

4 $$ \begin{gathered} \rho \,\left( {u\frac{\partial w}{{\partial r}} + w\frac{\partial w}{{\partial z}}} \right) = - \frac{\partial P}{{\partial z}} + \frac{\partial }{\partial z}\left( {2\mu (r,\,z)\frac{\partial w}{{\partial z}}} \right) + \frac{1}{r}\frac{\partial }{\partial r}\left( {r\,\mu (r,\,z)\left( {\frac{\partial u}{{\partial z}} + \frac{\partial w}{{\partial r}}} \right)} \right) \hfill \\ \quad \quad \quad \quad \quad \quad \quad \quad \quad - \left( {\sigma B_{0}^{2} + \frac{\mu (r,\,z)}{K}} \right)w + C^{ * } w^{2} , \hfill \\ \end{gathered} $$

5 $$ \begin{gathered} \left( {u\frac{\partial T}{{\partial r}} + w\frac{\partial T}{{\partial z}}} \right) = \frac{k}{{\rho \,c_{p} }}\,\left( {\frac{{\partial^{2} T}}{{\partial r^{2} }} + \frac{1}{r}\frac{\partial T}{{\partial r}} + \frac{{\partial^{2} T}}{{\partial z^{2} }}} \right) - \frac{1}{{\rho \,c_{p} \,r}}\frac{{\partial (rq_{r} )}}{\partial r}\, + \frac{{D_{B} K_{T} }}{{c_{p} c_{s} }}\nabla^{2} C \hfill \\ \quad \quad \quad \quad \quad \quad \quad \quad + D_{T} \,\left( {\nabla T} \right)^{2} + D_{B} \,\left( {\nabla T} \right)\left( {\nabla C} \right) + Q_{0} \,\left( {T - T_{2} } \right), \hfill \\ \end{gathered} $$

6 $$ u\frac{\partial C}{{\partial r}} + w\frac{\partial C}{{\partial z}} = D_{B} \left( {\frac{{\partial^{2} C}}{{\partial r^{2} }} + \frac{1}{r}\frac{\partial C}{{\partial r}} + \frac{{\partial^{2} C}}{{\partial z^{2} }}} \right) + \frac{{D_{T} }}{{T_{2} }}\left( {\frac{{\partial^{2} T}}{{\partial r^{2} }} + \frac{1}{r}\frac{\partial T}{{\partial r}} + \frac{{\partial^{2} T}}{{\partial z^{2} }}} \right) - A(C - C_{2} ). $$

The boundary conditions are defined as follows:

7 $$ u = 0,\;\;w^{\prime} = - c,\;\;T = T_{1} \,,\;\;C = C_{1} \;\;{\text{at}}\;\;r = r_{1} , $$

8 $$ u = - \frac{\partial H}{{\partial z}},\;\;w = - \beta \frac{\partial w}{{\partial r}},\;\;T = T_{2} ,\;\;C = C_{2} \;\;{\text{at}}\;\;r = r_{2} . $$

The radiative heat flux is given by the Rosseland approximation^11^:

9 $$ q_{r} = \frac{{ - 4\sigma^{ * } }}{{3k_{R} }}\frac{{\partial T^{4} }}{\partial r}. $$

*T*^4^ can be expressed as a linear function of temperature due to the small temperature differences within the flow. By expanding *T*^4^ in a Taylor series about *T*_2_ and ignoring higher-order terms, one obtains:

10 $$ T^{4} \approx 4T_{2}^{3} \,T - 3T_{2}^{4} . $$

Nanofluid's thermal-physical characteristics are defined by^37^:

11 $$ \mu_{nf} = \mu_{f} \,(1 - \varphi )^{ - 2.5} ,\,\;\rho_{nf} = \rho_{f} \,(1 - \varphi ) + \rho_{s} \,\,\varphi ,\,\;(\rho c_{p} )_{nf} = \,(\rho c_{p} )_{f} \,(1 - \varphi ) + \,(\rho c_{p} )_{s} \,\,\varphi , $$

12 $$ (\rho \beta_{T} )_{nf} = \,(\rho \beta_{T} )_{f} \,(1 - \varphi ) + \,(\rho \beta_{T} )_{s} \,\,\varphi ,\,\;(\rho \beta_{C} )_{nf} = \,(\rho \beta_{C} )_{f} \,(1 - \varphi ) + \,(\rho \beta_{C} )_{s} \,\,\varphi , $$

13 $$ D_{nf} = D_{f} \,(1 - \varphi ),\,\;{\raise0.7ex\hbox{${k_{nf} }$} \!\mathord{\left/ {\vphantom {{k_{nf} } {k_{f} }}}\right.\kern-0pt} \!\lower0.7ex\hbox{${k_{f} }$}} = {\raise0.7ex\hbox{${\left( {k_{s} + 2k_{f} - 2\varphi (k_{s} - k_{f} )} \right)}$} \!\mathord{\left/ {\vphantom {{\left( {k_{s} + 2k_{f} - 2\varphi (k_{s} - k_{f} )} \right)} {\left( {k_{s} + 2k_{f} + 2\varphi (k_{s} - k_{f} )} \right)}}}\right.\kern-0pt} \!\lower0.7ex\hbox{${\left( {k_{s} + 2k_{f} + 2\varphi (k_{s} - k_{f} )} \right)}$}}. $$

For the flow, the following are the suitable non-dimensional variables:

14 $$ \begin{gathered} r^{ * } = \frac{r}{d}\,\,,\,\;z^{ * } = \frac{z}{\lambda },\;u^{ * } = \frac{\lambda }{c\,d}u\,,\;w^{ * } = \frac{w}{c}\,,\;P^{ * } = \frac{{d^{2} }}{{\lambda c\mu_{0} }}P,\;T^{*} = \frac{{T - T_{2} }}{{T_{1} - T_{2} }}\,, \hfill \\ \,\delta = \frac{d}{\lambda }\,,\,\;C^{*} = \frac{{C - C_{2} }}{{C_{1} - C_{2} }}\,,\;t^{ * } = \frac{c}{\lambda }t\,,\;h = \frac{H}{d}\,,\;\varepsilon = \frac{b}{d},\,\;{\text{Re}} = \frac{\rho \,c\,d}{{\mu_{0} }},\,\;\mu_{f}^{*} = \frac{{\mu_{f} }}{{\mu_{0} }}. \hfill \\ \end{gathered} $$

Equations (2)–(6) become simpler when considering these variables, the star mark is removed for convenience, and low-Reynolds number approximations are used.

15 $$ \frac{\partial u}{{\partial r}} + \frac{u}{r} + \frac{\partial w}{{\partial z}} = 0, $$

16 $$ \frac{\partial P}{{\partial r}} = 0, $$

17 $$ \frac{\partial P}{{\partial z}} = \frac{1}{r}\frac{\partial }{\partial r}\left( {r\,\mu (r,\,z)\frac{\partial w}{{\partial r}}} \right) - \left( {M^{2} + \frac{\mu (r,\,z)}{{Da}}} \right)w - \,\mathop F\nolimits_{S} \,\,\mathop w\nolimits^{2} , $$

18 $$ \left( {\frac{3 + 4\,R}{{3\Pr }}} \right)\,\left( {\frac{{\partial^{2} T}}{{\partial r^{2} }} + \frac{1}{r}\frac{\partial T}{{\partial r}}} \right) + Nt\,\left( {\frac{\partial T}{{\partial r}}} \right)^{2} + Nb\,\left( {\frac{\partial T}{{\partial r}}\,\frac{\partial C}{{\partial r}}} \right)\, + D_{f} \left( {\frac{{\partial^{2} C}}{{\partial r^{2} }} + \frac{1}{r}\frac{\partial C}{{\partial r}}} \right) + Q_{0} \,T = 0, $$

19 $$ \left( {\frac{{\partial^{2} C}}{{\partial r^{2} }} + \frac{1}{r}\frac{\partial C}{{\partial r}}} \right) + \frac{Nt}{{Nb}}\,\left( {\frac{{\partial^{2} T}}{{\partial r^{2} }} + \frac{1}{r}\frac{\partial T}{{\partial r}}} \right) - \delta \,\mathop C\nolimits^{m} = 0. $$

The following non-dimensional boundary conditions imply that the fluid particles closest to the solid boundary of the inner tube are traveling equal displacements at equal times in the opposite direction. In addition, the solid boundary of the inner tube is kept at zero temperature and zero nanoparticles concentration. While, at the boundary of the outer tube, both temperature and nanoparticles concentration are kept at unity, furthermore, the fluid velocity will not equal zero relative to the solid boundary.

Hence, the boundary conditions (7) and (8) are changed from their dimensionless form to:

20 $$ w = - 1\,,\;\;T = C = 0,\;\;{\text{at}}\;\;r = \varepsilon . $$

21 $$ w = - \beta \frac{\partial w}{{\partial r}},\;\;T = C = 1,\;\;{\text{at}}\;\;r = h\, = 1 + \varepsilon \sin \,2\pi z. $$

We take into account the fluid's viscosity depending on radial and axial velocity components^34^:

22 $$ \mu (r,\,z) = 1 - \alpha r/h. $$

Equations (17)–(19) are extremely non-linear ordinary differential equations. If $D_{a} \to \infty$, $\mathop {{\text{M = }}F}\nolimits_{S} = 0$ and in the absence of heat transfer and nanoparticles concentration, this study tends to the work of Eldabe et al.^34^.

## Method of solution

### Homotopy perturbation method

The above system of differential Eqs. (17)–(19) that governs the problem is highly non-linear and complicated. So, the homotopy perturbation technique is used to obtain a semi-analytical solution for ordinary and partial differential equations. In addition, it is a combination of the perturbation method and the homotopy analysis method. One of the most important steps in the homotopy perturbation method is to guess an initial solution.

Following^12^, Eqs. (17), (18), and (19) can be written as follows:

23 $$ H\,(p,\,w) = \,L(w) - L(w_{10} ) + pL(w) - \frac{p}{1 - (\alpha /h)\,r}\,\left( {(\alpha /h)\frac{\partial w}{{\partial r}} + \frac{\partial P}{{\partial z}}} \right.\left. { + \left( {M^{2} + \frac{1 - (\alpha /h)\,r}{{Da}}} \right)w + \,\mathop F\nolimits_{S} \,\,\mathop w\nolimits^{2} } \right), $$

24 $$ H\,(p,\,T) = \,L(w) - L(w_{10} ) + p\,L(w_{10} ) + p\left( {\frac{3\,\Pr }{{3 + 4\,R}}} \right)\,\left( {Nb\,\left( {\frac{\partial T}{{\partial r}}\,\frac{\partial C}{{\partial r}}} \right) + Nt\,\left( {\frac{\partial T}{{\partial r}}} \right)^{2} } \right.\left. { + Q_{0} \,T + D_{f} \left( {\frac{{\partial^{2} C}}{{\partial r^{2} }} + \frac{1}{r}\frac{\partial C}{{\partial r}}} \right)} \right), $$

25 $$ H\,(p,\,C) = \,L(C) - L(C_{10} ) + pL(C) + p\,\left( {\frac{Nt}{{Nb}}\,\left( {\frac{{\partial^{2} T}}{{\partial r^{2} }} + \frac{1}{r}\frac{\partial T}{{\partial r}}} \right) - \delta \,\mathop C\nolimits^{m} } \right). $$

With the linear operator. The initial guesses for $w_{10}$, $T_{10}$ and $C_{10}$ are:

26 $$ w_{10} = \frac{{\ln \,r - \ln \,r_{1} }}{{\ln \,r_{2} - \ln \,r_{1} + (\beta /r_{2} )}} - 1,\;\;T_{10} = C_{10} = \frac{{\ln \,r - \ln \,r_{1} }}{{\ln \,r_{2} - \ln \,r_{1} }}. $$

Then, we assume that:

27 $$ (\,T,\,C) = (T_{0} ,\,C_{0} ) + p\,(\,T_{1} ,\,C_{1} ) + p^{2} \,(\,T_{2} ,\,C_{2} ) + \cdots . $$

Substituting from (27) into (23), (24), and (25), we obtain when p = 1:

28 $$ \begin{gathered} w(r,\,z) = \frac{{\ln \,r - \ln \,r_{1} }}{{\ln \,r_{2} - \ln \,r_{1} + (\beta /r_{2} )}} - 1 + \left( {a_{1} \,\ln \,r + a_{2} \,(\ln \,r)^{2} } \right)\,r + \left( {a_{3} \, + a_{5} \,\ln \,r + a_{6} (\ln \,r)^{2} } \right) \hfill \\ \quad \quad \quad \quad \quad \times Li_{2} \left( {a_{4} \,r} \right) + \,(a_{7} + a_{8} \,(\ln \,r))\,Li_{3} \left( {a_{4} \,r} \right) + a_{9} \,Li_{4} \left( {a_{4} \,r} \right) + a_{10} , \hfill \\ \end{gathered} $$

29 $$ T(r,\,z) = \frac{{\ln \left( {r/r_{1} } \right)}}{{\ln \left( {r_{2} /r_{1} } \right)}} + a_{11} \,r^{2} + a_{12} \,\left( {\ln r} \right) + a_{12} \,r^{2} \left( {\ln r} \right) + a_{13} \,\left( {\ln r} \right)^{2} , $$

30 $$ C(r,\,z) = \frac{{\ln \left( {r/r_{1} } \right)}}{{\ln \left( {r_{2} /r_{1} } \right)}} + a_{14} + a_{15} \ln \,r - \frac{Nt}{{Nb}}\,T_{1} (r,\,z), $$

where $Li_{n} \left( z \right)$ is the polylogarithm function, which is defined by:

31 $$ Li_{n} \left( z \right) = \frac{{( - 1)^{n - 2} }}{(n - 2)!}\,\int\limits_{0}^{1} {(\ln \,(t))^{n - 2} } \,\ln \,(1 - zt)\,dt, $$

The constants are available upon request from the authors. In the part after this, the results will be discussed.

### Numerical treatment

In order to validate the obtained values in the previous method, we solve the above system of non-linear differential Eqs. (17)–(19) numerically.

Let $\,w = Y_{1} ,\,T = Y_{3} ,\,C = Y_{5}$. Hence, Eqs. (14)–(16) can be written as follows:

32 $$ Y^{\prime}_{{1}} = Y_{{2}} ,\,Y^{\prime}_{{2}} + \frac{1}{r}Y_{{2}} = \frac{ - p}{{1 - (\alpha /h)\,r}}\,\left( {(\alpha /h)\,Y_{{2}} + \frac{\partial P}{{\partial z}} + \left( {M^{2} } \right. + \,\left. {\left. {\frac{1 - (\alpha /h)\,r}{{Da}}} \right)\,Y_{1} + \,\mathop F\nolimits_{S} \,\,Y_{1}^{2} } \right)} \right., $$

33 $$ Y^{\prime}_{3} = Y_{4} ,\,Y^{\prime}_{4} + \frac{1}{r}Y_{4} = \,\left( {\frac{ - 3\,\Pr }{{3 + 4\,R}}} \right)\,\left( {Nb\,Y_{4} \,Y_{6} + Nt\,Y_{4}^{2} + + Q_{0} \,Y_{3} + D_{f} \left( {Y^{\prime}_{6} + \frac{1}{r}Y_{6} } \right)} \right), $$

34 $$ Y^{\prime}_{5} = Y_{6} ,\,Y^{\prime}_{6} = - \frac{Nt}{{Nb}}\,\left( {Y^{\prime}_{4} + \frac{1}{r}Y_{4} } \right) + \delta \,Y_{5}^{m} , $$

where prime denotes differentiation with respect to r, and this system (29)–(31) is subject to the boundary conditions:

35 $$ Y_{1} = - 1,\;\;Y_{3} = Y_{5} = 0,\;\;{\text{at}}\;\;r = \varepsilon . $$

36 $$ Y_{1} = - \beta \,Y_{2} \,,\;\;Y_{3} = Y_{5} = 1,\;\;{\text{at}}\;\;r = h\, = 1 + \varepsilon \sin \,2\pi z. $$

The system of Eqs. (32)–(34), with the boundary conditions (35) and (36), are more complex to handle as supplemental nonlinear terms appear in the equations of motion. So, we apply the shooting technique by using the NAG Fortran library, namely the subroutine D02HAF, which requires the guessing of starting values for missing initial and terminal conditions. The Rung-Kutta-Merson-Kutta-Mersonvariable step size is used in this subroutine in order to control the local truncation error, and then the modified Newton–Raphson technique is applied to make successive corrections to the estimated boundary values. The process is repeated iteratively until convergence is obtained, i.e., until the absolute value of the difference between every two successive approximations of the missing conditions is less than ε (in our spacing, ε = 10^−5^). Furthermore, we have compared the obtained values with those obtained by HPM, as shown in Table 2.

**Table 2:** Comparison between numerical method and HPM for the velocity, temperature and nanoparticles concentration.

r	The velocity *w*		The temperature T		The nanoparticles concentration C	
	HPM	Numerical method	HPM	Numerical method	HPM	Numerical method
0.3	− 1	− 1	0	0	0	0
0.6	0.85786	0.83903	2.71089	2.60909	− 0.14999	− 0.15109
0.9	1.25470	1.20829	3.40220	3.47313	− 0.04723	− 0.05091
1.2	0.96019	1.00092	2.73329	2.69230	0.34709	0.35093
1.5	0.37019	0.37019	1	1	1	1

## Results and discussion

Here, the variation of slip condition parameters and the coefficient of viscosity concerning both radial coordinate *r* and axial coordinate *z* are analyzed. Also, long wavelength and small Reynolds number assumptions are assumed in this work. The default values of problem-related parameters are taken as:

$$ Pz = - 1, \alpha = 0.15, \beta = 0.3, M = 5, Da = 0.1, Fs = 5, R = 1, Q_{0} = 10, Pr = 1.5, Nt = 3.5, Nb = 1.5, r1 = 0.3, z = 0.8, \varepsilon = 0.1. $$

In the same manner as in the prior work^11^, the following human small intestine parameter values are utilized:

d = 1.2 cm,c = 2 cm/min, λ = 8.1 cm.

### Velocity profile

**Figure 2:**
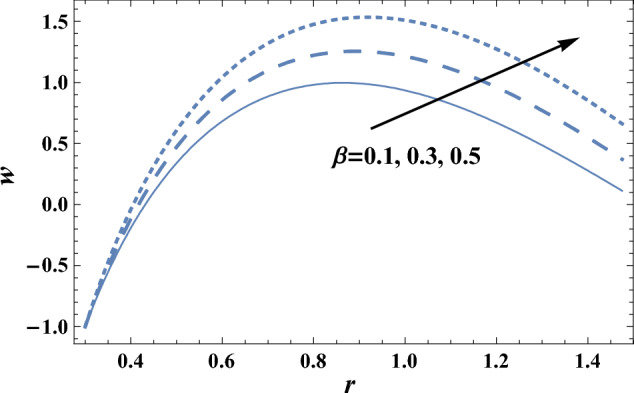
The axial velocity w is plotted with r, for different values of $\beta$.

**Figure 3:**
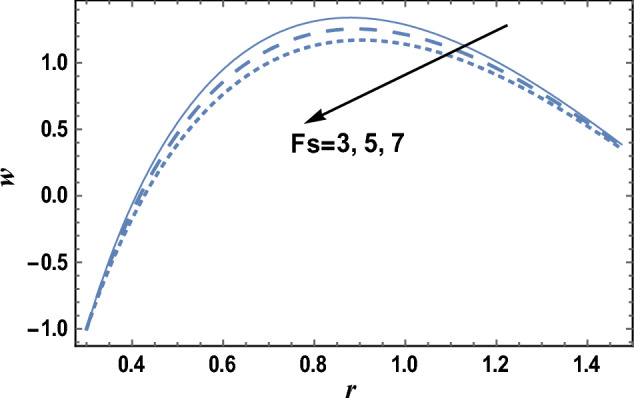
The axial velocity w is plotted with r, for different values of Fs.

**Figure 4:**
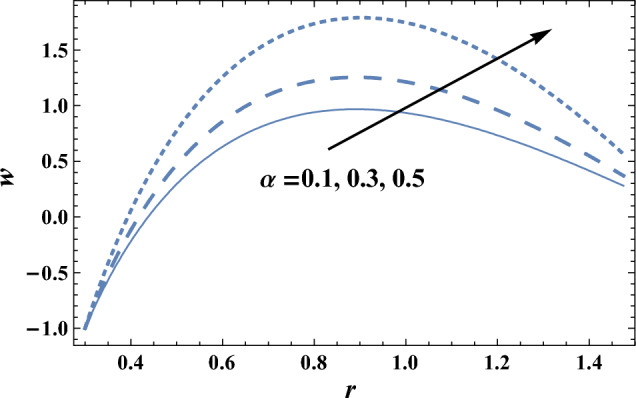
The axial velocity w is plotted with r, for different values of $\alpha$.

**Figure 5:**
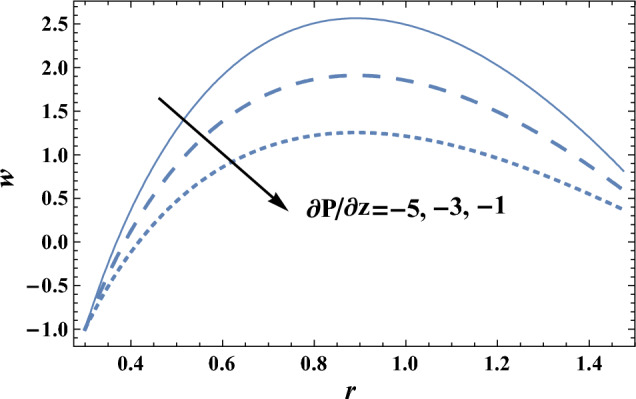
The axial velocity w is plotted with r, for different values of $\partial P/\partial z$.

The effects of the slip velocity parameter $\beta$ and Forchheimer number Fs on the axial velocity *w,* which is a function of the radial coordinate *r,* are shown in Figs. 2 and 3, and it is shown that the axial velocity *w* increases as $\beta$ increases, while it decreases as Fs increases. It is also noted that for each value of both $\beta$ and Fs, there exists a maximum value of *w*, whose value increases by increasing $\beta$ and decreases by increasing Fs, and all maximum values occur at r = 0.83. The following clarifies the result in Fig. 2: due to the difference between the particle velocity and the undisturbed velocity of the fluid at the same particle position, it is found that the increment of the slip viscosity parameter will help the fluid to move easier. Figures 4 and 5 show the influence of the viscosity parameter $\alpha$, and the pressure gradient $\partial P/\partial z$ on the axial velocity* w*, respectively. It is noted from these figures that the axial velocity increases with the increase of $\alpha$, whereas it decreases as $\partial P/\partial z$ increases. Now, we will clarify the result in Fig. 4, The viscosity parameter is inversely proportional to the dynamical viscosity, according to Eq. (19). It is well known that the fluid particles motion becomes slow when the viscosity of the fluid increases. Thus, this will help the viscosity parameter to increase the fluid velocity. Similarly, if we draw the variation of *w* with *r* for different values of Darcy number Da, we will get a figure with the same curve behavior as in Fig. 3; except that the obtained curves are very close to those obtained in Fig. 3; but this figure will not be given there to save space. Furthermore, this result is due to the fact that as the Darcy number increases, the convective mode becomes stronger due to an increase in the permeability of the medium. So, the fluid's axial velocity increases.

### Heat characteristics

**Figure 6:**
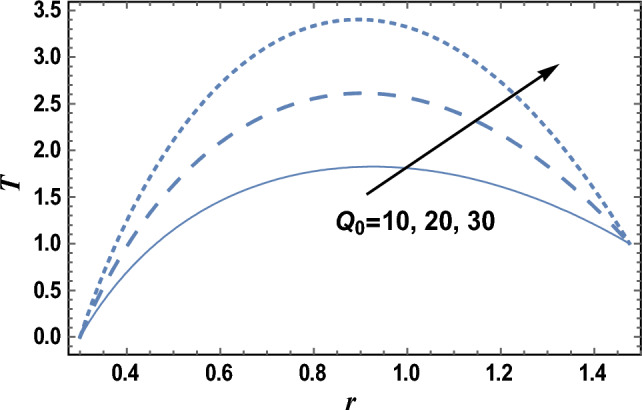
The temperature distribution T is plotted with r, for different values of Q_0_.

**Figure 7:**
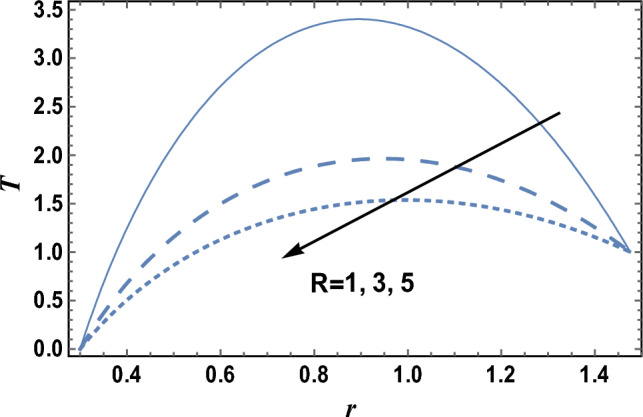
The temperature distribution T is plotted with r, for different values of R.

**Figure 8:**
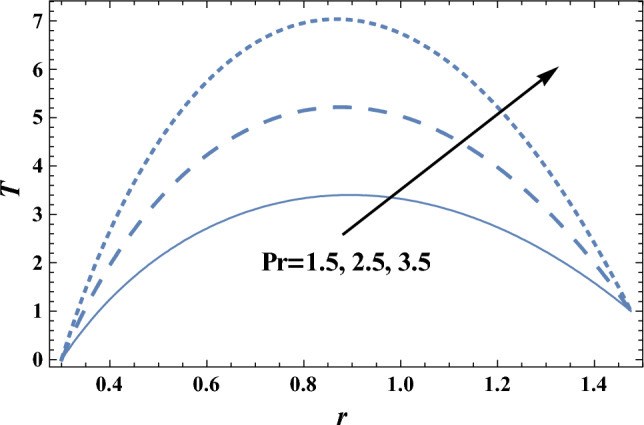
The temperature distribution T is plotted with r, for different values of Pr.

Heat is the motion total energy of the material molecules, whereas temperature refers to the average energy measure of the motions of these molecules in the material. The heat is dependent on many factors, including the speed, size, and number of particles. The effects of the heat source parameter Q_0_ and radiation parameter R on the temperature distribution T, which is a function of *r,* are shown in Figs. 6 and 7, respectively. It is clear from these figures that the temperature distribution is always positive, and it increases by increasing Q_0_, while it decreases as R increases. It is also noted from Figs. 6 and 7 that T increases as r increases until a maximum value of T, after which T decreases. Similar outcomes can be obtained, as shown in Fig. 6; by drawing* T* versus *r* for various values of both the thermophoresis parameter Nt and the Brownian motion parameter Nb, but the figure is not given here to save space. The result in Fig. 7; agrees with the physical expectation and previous definition in the beginning and with those obtained by^11^. Figure 8; shows the variation of the temperature distribution T with r for various values of Prandtl number Pr. It is seen from Fig. 8; that the obtained curves of temperature are the same as those obtained in Fig. 6. Now, we will explain the previous result physically. In problems of heat transfer, the Prandtl number dominates the momentum relative thickness and thermal boundary layers. So, when Pr is high, it means that heat transfer is more likely to occur by the momentum of the fluid than by thermal diffusion.

### Nanoparticles concentration characteristics

**Figure 9:**
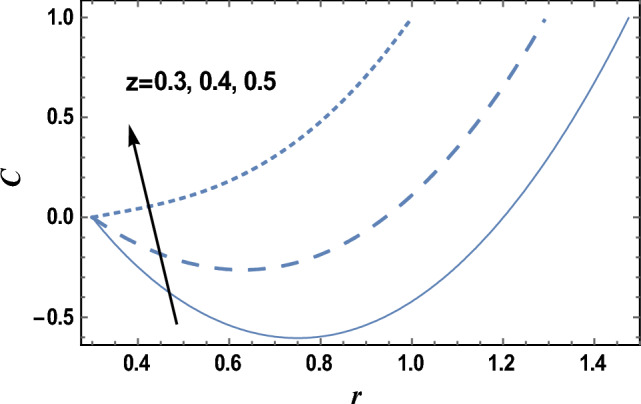
The nanoparticles concentration C is plotted with r, for different values of z.

**Figure 10:**
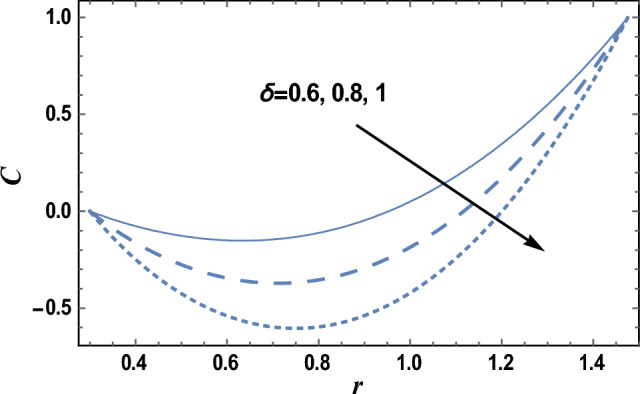
The nanoparticles concentration C is plotted with r, for different values of δ.

Equation (27) evaluates how the nanoparticles concentration distribution C changes with the radial coordinate r. Figures 9 and 10 show the influence of axial coordinate z and the chemical reaction parameter δ on the nanoparticle’s concentration distribution C, respectively. It is found that the nanoparticles concentration increases with increasing z but decreases with increasing δ. Furthermore, the nanoparticles concentration is always negative near the inner tube and positive near the outer tube, and it decreases as r increases and reaches a minimum value (at a finite value of r: r = r_0_), after which it increases. The effects of other parameters are like those obtained in Figs. 9 and 10. Now, we will explain the obtained result in Fig. 9; The axial coordinate is sometimes called the height and is the signed distance from the chosen plane to a definite point. From Eq. (19); the fluid viscosity is directly proportional to the axial coordinate. It is well known that viscosity enhances the consistency of fluid particles and nanoparticles; consequently, nanoparticles concentration will increase. Furthermore, according to the problem model, there are relaxations and contractions in the axial direction. These relaxations and contractions are generated along the axial direction, which helps increase the concentration of nanoparticles. Furthermore, the result in Fig. 10; is due to the following: when more particles are present in each amount of space, a greater number of collisions will naturally occur between those particles. This will reduce the interface between particles and, consequently, the concentration of nanoparticles.

### Skin friction profiles

Table 3 presents numerical results for the skin friction at the outer tube $\tau$ for various values of the viscosity parameter $\alpha$ and the slip velocity parameter $\beta$. It is found from Table 3) that an increase in $\alpha$ increases the values of $\tau$. While an increase in $\beta$ causes a decrease in $\tau$. Moreover, the results in Table 3 agree with those obtained by^35^.

**Table 3:** Values of $\tau$ for various values of $\alpha$ and $\beta$.

$\alpha$	$\beta$	$\tau$
0.1	0.1	2.2680
0.2	0.1	2.5794
0.3	0.1	2.9999
0.3	0.2	1.2567
0.3	0.3	1.2489

### Nusselt and Sherwood numbers profiles

The behavior of both the Nusselt number Nu and the Sherwood number $Sh$ with the thermophoresis parameter Nt, the Brownian motion parameter Nb, and the chemical reaction parameter δ is presented in Table 4. It is clear from Table 4. that the Sherwood number $Sh$ increases with increasing both Nt and δ, while it decreases with increasing Nb. Moreover, it is noted that when both Nt and Nb increase, the Nusselt number decreases, but it increases as δ increases. The results in this table agree with the fact that when the volume of nanoparticles is small (e.g., spherical carbon nanotubes, cylindrical graphene, platelet copper), a higher surface temperature can cause a large rise in Nusselt number proportionate to heat transfer when ternary hybrid nanofluid flows over an impermeable, moveable wall^40^. In this case, our results can be considered a comparison between nanofluid and ordinary fluid (Nt = Nb = 0).

**Table 4:** Values of Nusselt number $Nu$ and Sherwood number Sh for various values of $\alpha$ and $\beta$ in both cases of ordinary and nanofluid.

*Nt*	*Nb*	$\delta$	Nu	Sh	
3.5	1.5	1.0	− 2.0709	1.0614	Nanofluid
2.0	1.5	1.0	− 1.6774	1.0560	
0.5	1.5	1.0	− 1.2837	1.0530	
0.5	1.0	1.0	− 1.1525	1.0532	
0.5	0.5	1.0	− 0.9783	1.0534	
0.0	0.0	0.6	− 0.9971	0.8952	Ordinary fluid
0.0	0.0	0.2	− 1.0214	0.7589	

## Conclusion

This study aims to analyze the effects of both slip velocity conditions and radially varying viscosity on the peristaltic flow of copper oxide Cu O nanofluid between two co-axial cylinders. The fluid flows through a non-Darcy porous medium in the presence of a heat source, a chemical reaction, and a uniform magnetic field. The homotopy perturbation technique is used to obtain a semi-analytical solution to the governing equations. The present analysis can serve in medical applications, engineering, and industry^41,42^. In this work, blood is considered a base fluid containing the nanoparticles of Cu O. Furthermore, investigations of these effects together are very useful due to their important vital applications in various scientific fields, especially in medicine and medical industries, such as endoscopes, respirators, and diverse medical implementations, as nanoparticles can be utilized in the remedy of cancer tumors. Moreover, this study investigates the influence of an endoscope on the unsteady, incompressible, flow which plays a very important role in medical diagnosis due to its wild clinical applications in determining the reasons behind many diseases in the human organs. For example, the motion of gastric juice when an endoscope is inserted through a small intestine. The effects of various problem parameters on the flow are discussed through numerical computations. The main results can be abbreviated as follows:

1. The axial velocity *w* increases with the increase of each slip velocity parameter $\beta$ and the viscosity parameter $\alpha$, whereas it decreases as both the Forchheimer number Fs, Darcy number Da and the pressure gradient $\partial P/\partial z$ increase.
2. All obtained curves of the velocity for different values of $\partial P/\partial z$, $\beta$, $\alpha$, Fs, and *Da* coincide near the inner tube and don’t intersect at the outer tube; moreover, the axial velocity increases as r increases till a maximum value, after which it decreases.
3. The temperature increases with the increase in each of the heat source parameter Q0 and Prandtl number Pr, whereas it decreases as the radiation parameter R increases.
4. By increasing the radial coordinate r, the temperature T for different values of the problem physical parameters becomes greater and ends up at a maximum value at a finite value near the outer tube.
5. The nanoparticles concentration C has an opposite behavior compared to the temperature behavior. This agrees with nature.

## Applications

Although peristaltic pumps have many applications, such as adhesives for cement, ultrafiltration, and the transfer of fuels and lubricants, they have advantages and disadvantages (“Supplementary information”).

### Advantages

1. It has excellent reliability throughout the day and is unaffected by the weather.
2. The transportation distance is low, and the pipeline can make quick cuts.
3. Significant environmental advantages and the absence of contaminants.
4. It uses the least amount of energy compared to other forms of transportation.
5. Low cost, no pollution, safe, and dependable.

### Disadvantages

1. Less efficient than other artificial lift techniques.
2. Production rates are often doubled for power fluid injection rates.
3. Space restrictions, particularly for installations offshore.

### Supplementary Information

Supplementary Information.

## Data Availability

The datasets generated and/or analyzed during the current study are not publicly available due to [all the required data are only with the corresponding author] but are available from the corresponding author on reasonable request.

## List of symbols

A: Non-dimensional chemical reaction parameter
*a*: The radius of the inner tube (L)
*B*: The magnetic field $= (B_{0} ,0,0)$
*b*: The dimensional wave amplitude
$C^{ * }$: Non-Darcian parameter
$\mathrm{c}$: The propagation velocity along z-direction
*c*_*p*_: The specific heat at constant pressure
*C*: The fluid nanoparticles concentration (M)
*C*_1_: Nanoparticles concentration at $r={r}_{1}$ (M)
*C*_2_: Nanoparticles concentration at $r={r}_{2}$ (M)
*d*: The uniform radius of the outer tube
Da: Darcy number $= \frac{K}{{d^{2} }}$
*D*_B_: Brownian diffusion coefficient
*D*_*T*_: Thermophoretic diffusion coefficient
Ec: $${\text{Eckert}}\;{\text{number}} = \frac{{c^{2} }}{{c_{P} (T_{1} - T_{2} )}}$$
Fs: Forchheimer number $= d\,C^{*}$
*k*: Coefficient of thermal conductivity
*K*: The permeability parameter
*M*: Magnetic field parameter $= \frac{{\sigma B_{0}^{2} d^{2} }}{{\mu_{0} }}$
*N*_*b*_: $$ {\text{Brownian}}\;{\text{motion}}\;{\text{parameter}} = \frac{{D_{B} \,(C_{1} - C_{2} )\,\left( {\rho c} \right)_{p} }}{{\left( {\rho c} \right)_{f} \,c\,d}} $$
*N*_*t*_: $$ {\text{The}}\;{\text{thermophoresis}}\;{\text{parameter}} = \frac{{D_{T} \,(T_{1} - T_{2} )\,\left( {\rho c} \right)_{p} }}{{T_{1} \,\left( {\rho c} \right)_{f} \,c\,d}} $$
*P*: The fluid pressure
P_r_: Prandtl number $$= \frac{{\mu_{0} \,c_{P} }}{k}$$
Q_0_: Heat source parameter
${q}_{r}$: The radiative heat flux
*r*: Radial coordinate (L)
*R*: Radiation parameter $$= \frac{{4\sigma^{ * } T_{w}^{3} }}{{k\,k_{R} }}$$
Re: Reynolds number $= \frac{\rho \,c\,d}{{\mu_{0} }}$
*t*: The time (t)
*T*: The fluid temperature
*T*_1_: The temperature at $r={r}_{1}$
*T*_2_: The temperature at $r={r}_{2}$
*u*: Radial velocity (L t^−1^)
*w*: Axial velocity (L t^−1^)
*z*: Axial coordinate (L)

## Greek symbols

$\alpha$: The viscosity parameter
*σ*: The electrical conductivity
*ε*: The dimensionless wave amplitude
λ: The wavelength
$\mu$: The viscosity of the fluid
$\mu_{0}$: The fluid viscosity at $r={r}_{1}$
$\rho$: The fluid density
$(\rho c)_{p}$: Effective heat capacity of the nanoparticle material
