# Supplementary material for: Chemically reacted blood Cu O nanofluid flow through a non-Darcy porous media with radially varying viscosity
Source: Sci Rep. 2024 Jan 18;14:1650. doi: 10.1038/s41598-023-48692-3 (PMC10796964; doi:10.1038/s41598-023-48692-3)
Supplement: Supplementary file 1 — Supplementary Information. [file 41598_2023_48692_MOESM1_ESM.docx]

**Appendix**

**Substituting from (11) in (1-b), we get**

$d r_{2}^{*}=d+b\sin\frac{2\pi}{\lambda} \lambda z^{*}, z=Z-ct$, then divide by d and omit star mark

$$r_{2}=1+\varepsilon\sin2\pi z$$
